# The African Crane Database (1978-2014): Records of three threatened crane species (Family: Gruidae) from southern and eastern Africa

**DOI:** 10.3897/BDJ.4.e9794

**Published:** 2016-09-14

**Authors:** Tanya Smith, Samantha Page-Nicholson, Kerryn Morrison, Bradley Gibbons, M. Genevieve W. Jones, Mark van Niekerk, Bronwyn Botha, Kirsten Oliver, Kevin McCann, Lizanne Roxburgh

**Affiliations:** ‡ International Crane Foundation / Endangered Wildlife Trust Partnership, Johannesburg, South Africa; § Endangered Wildlife Trust, Johannesburg, South Africa; | Percy FitzPatrick Institute of African Ornithology, DST/NRF Centre of Excellence, University of Cape Town, Cape Town, South Africa; ¶ Jean Monnet University, Saint Etienne, France; # Flower Valley Conservation Trust, Gansbaai, South Africa; ¤ Wildlands Conservation Trust, Hilton, South Africa

**Keywords:** Sightings, occurrence, distribution, *Anthropoides paradiseus*, *Balearica regulorum*, *Bugeranus carunculatus*, Africa, Aves

## Abstract

**Background:**

The International Crane Foundation (ICF) / Endangered Wildlife Trust’s (EWT) African Crane Conservation Programme has recorded 26 403 crane sightings in its database from 1978 to 2014. This sightings collection is currently ongoing and records are continuously added to the database by the EWT field staff, ICF/EWT Partnership staff, various partner organizations and private individuals. The dataset has two peak collection periods: 1994-1996 and 2008-2012. The dataset collection spans five African countries: Kenya, Rwanda, South Africa, Uganda and Zambia; 98% of the data were collected in South Africa. Georeferencing of the dataset was verified before publication of the data. The dataset contains data on three African crane species: Blue Crane *Anthropoides paradiseus*, Grey Crowned Crane *Balearica regulorum* and Wattled Crane *Bugeranus carunculatus*. The Blue and Wattled Cranes are classified by the IUCN Red List of Threatened Species as Vulnerable and the Grey Crowned Crane as Endangered.

**New information:**

This is the single most comprehensive dataset published on African Crane species that adds new information about the distribution of these three threatened species. We hope this will further aid conservation authorities to monitor and protect these species. The dataset continues to grow and especially to expand in geographic coverage into new countries in Africa and new sites within countries. The dataset can be freely accessed through the Global Biodiversity Information Facility data portal.

## Introduction

The Gruidae family, which includes 15 living species of cranes on five continents (Harris and Mirande 2013), is among the most threatened bird families worldwide (Meine and Archibald 1996). Threats to cranes are greatest in Asia and sub-Saharan Africa, where they are affected by the impacts of large and expanding human populations, such as habitat loss, land degradation and land use changes, that are exacerbated by poorly integrated environmental protection (Harris and Mirande 2013). Africa has six crane species: Blue Crane *Anthropoides paradiseus*, Wattled Crane *Bugeranus carunculatus*, Grey Crowned Crane *Balearica regulorum*, Demoiselle Crane *Anthropoides virgo*, Eurasian Crane *Grus grus* and Black Crowned Crane *Balearica pavonina* (Harris and Mirande 2013). This published dataset contains data about Blue, Grey Crowned and Wattled Cranes, which are the species that the ICF/EWT Partnership's African Crane Conservation Programme (ACCP) currently focuses it work on.

The Grey Crowned Crane is currently listed as Endangered as a result of very rapid population declines of up to 80% over the last three generations (BirdLifeInternational 2013). With between 26 500 and 33 500 individuals remaining, this species of crane occurs in the eastern and southern areas of the African continent and is threatened primarily by habitat loss, illegal trade and disturbance (Morrison 2015, van Niekerk 2010, Olupot et al. 2010).

The Blue Crane is confined to South Africa with the exception of a small, isolated population in northern Namibia. This species has the most restricted range of any crane species (Harris and Mirande 2013). The Blue Crane population is estimated at >25 000 individuals (McCann et al. 2007) and is thought to be fairly stable, if not increasing (Harris and Mirande 2013). The dominant threat to Blue Cranes in South Africa is collision with power-lines (Shaw et al. 2010). A study by Shaw et al. 2010 determined that 12% of the Blue Crane population in the Overberg region of the Western Cape, South Africa, is killed annually by power-lines.

The Wattled Crane is the most specialized of the African crane species as it depends on largely intact wetland and floodplain habitats. The Wattled Crane occurs in eastern and southern Africa with a stable to decreasing population estimated to be less than 8 000 individuals (Beilfuss et al. 2007).

Cranes are iconic species and are often used in many areas as flagship species to gain recognition and support for conservation and biodiversity efforts (Harris and Mirande 2013). In South Africa, the Blue Crane is the national bird while the Grey Crowned Crane is the national bird of Uganda. In Zambia, the Wattled Crane is the emblem of BirdWatch Zambia, the BirdLife partner in this country. It is vital that monitoring of these threatened species is continued to aid effective conservation and mitigation strategies. According to Morrison (2015), some of the most pressing knowledge gaps regarding Grey Crowned Cranes are national level population size estimates and knowledge about habitat requirements and the extent of (remaining) suitable habitat. The African Crane Database contains a variety of types of data on cranes, including sightings, breeding, characteristics of and threats to breeding sites, aerial surveys, satellite tracking, and mortality data, and sets the foundation for addressing these knowledge gaps. The publication of the sightings data from this database to the Global Biodiversity Information Facility (GBIF) data portal will hopefully encourage further contributions of crane data from across the African continent to the Crane Database, ultimately allowing for some of our knowledge gaps to be filled.

## General description

### Purpose

Between 1978 and 1995, data were collected in South Africa by multiple individuals and organisations including: the Transvaal Provincial Administration (now the Mpumalanga Parks and Tourism Authority), the Natal Parks Board (now Ezemvelo KwaZulu-Natal Wildlife), the Southern African Crane Foundation (now the KwaZulu-Natal Crane Foundation) the Overberg Crane Group and Cape Nature. Two projects related to cranes were set up by the Endangered Wildlife Trust (EWT): an Environmental Impact Assessment project for a power-line development in KwaZulu-Natal in 1994, and the Highlands Crane Group on the Mpumalanga Highlands in 1995. Also in 1995, the EWT’s South African Crane Working Group was established as an umbrella project to guide and direct crane conservation in the country. In 2005, through the expansion of the work into other countries in Africa and in partnership with the International Crane Foundation, the African Crane Conservation Programme (ACCP) was established. Data were (and still are) collected by staff of this partnership and by partner organisations, which include both governmental authorities and other NGOs. Data from these various sources were combined and developed into the African Crane Database, which is currently held at the EWT.

## Project description

### Title

A sightings database of crane species recorded in five African countries

### Funding

The data have been collected both voluntarily by individuals, and through projects set up for crane conservation which were funded through either corporates or international trusts and foundations. Since 1994, the major funders have been, in alphabetical order: Anglo American Chairman’s Fund, Critical Ecosystems Partnership Fund, Darwin Initiative, Disney Conservation Fund, Dohmen Family Foundation, Eskom, John D. and Catherine T. MacArthur Foundation, National Lottery Distribution and Trust Fund, Nestlé, PG Bison, Rand Merchant Bank, Rand Water, Whitley Fund for Nature and WWF Nedbank Green Trust.

## Sampling methods

### Study extent

The study covers five countries in Africa (Kenya, Rwanda, South Africa, Uganda and Zambia). South Africa is the best-represented country in terms of data, which is due to the fact that the EWT has had a full time crane conservation programme in South Africa since 1994, that merged with the ICF in 2005, to become the ACCP. The fewest sightings were recorded in Kenya. However, short term projects in Rwanda, Kenya and Uganda have been funded over the last 20 years, and as a result, crane records have increased.

### Sampling description

All sightings have been recorded on an ad hoc basis across the regions and projects. However, they were collected from areas where crane studies or conservation projects were being undertaken at the time. All reported sightings, with sufficient information to be meaningful, were captured opportunistically. Generally, sightings of cranes within this dataset are from roadside collections. For this reason, distance and direction from the location of the recorder have frequently been recorded in the dataset - this information is captured in the locationRemarks field. The sampling was often concentrated around the location where ICF / EWT Partnership field staff were based within project areas, but this also corresponds with the core regions for cranes.

### Quality control

The dataset has gone through a cleaning and georeferencing process to ensure GPS points and location information are accurate. (10 % of the data were removed through this process due to inaccurate GPS coordinates; missing locality information or if they generally lacked information for the observation to be meaningful. Taxonomic and vernacular names were checked for consistency in naming and any errors were corrected. Terms in the dataset are in accordance with those set by the Darwin Core (DwC) Standard (Darwin Core Task Group, 2009).

### Step description

Observations of crane species were incorporated into the dataset by the EWT and ICF/EWT Partnership employees, which included sightings that were reported by the general public as well as by EWT, ICF/EWT Partnership staff or partner organizations. Data were only included in the dataset if there was sufficient information (e.g. GPS coordinates, individual specifics, number of individuals seen etc.). Details of the sightings were recorded, which included: age class (adult, juvenile, or chick), number of individuals, their activity (breeding, feeding, flying, roosting) and group type (single, pair, single/mixed species flock or family). All coordinates were converted to decimal degrees, datum WGS84, if not provided by the reporter in decimal degrees. Other location details were also recorded (country, province/district, and specific locality).

## Geographic coverage

### Description

**Figure 1.**
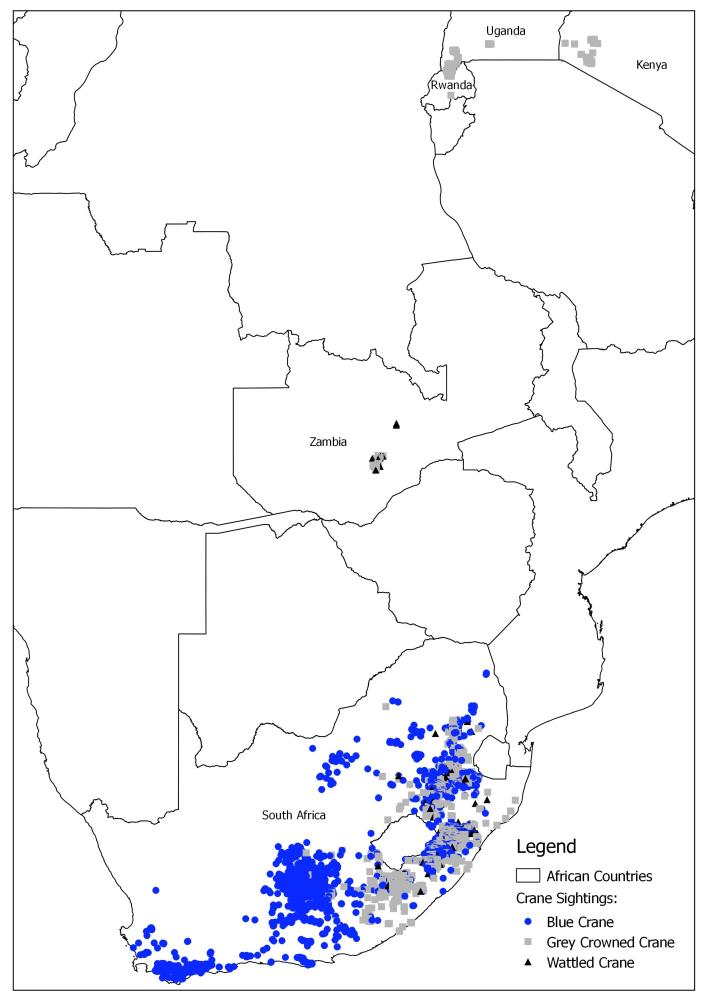
Distribution of crane sightings (1978-2014) of three crane species in Africa.

**Figure 2.**
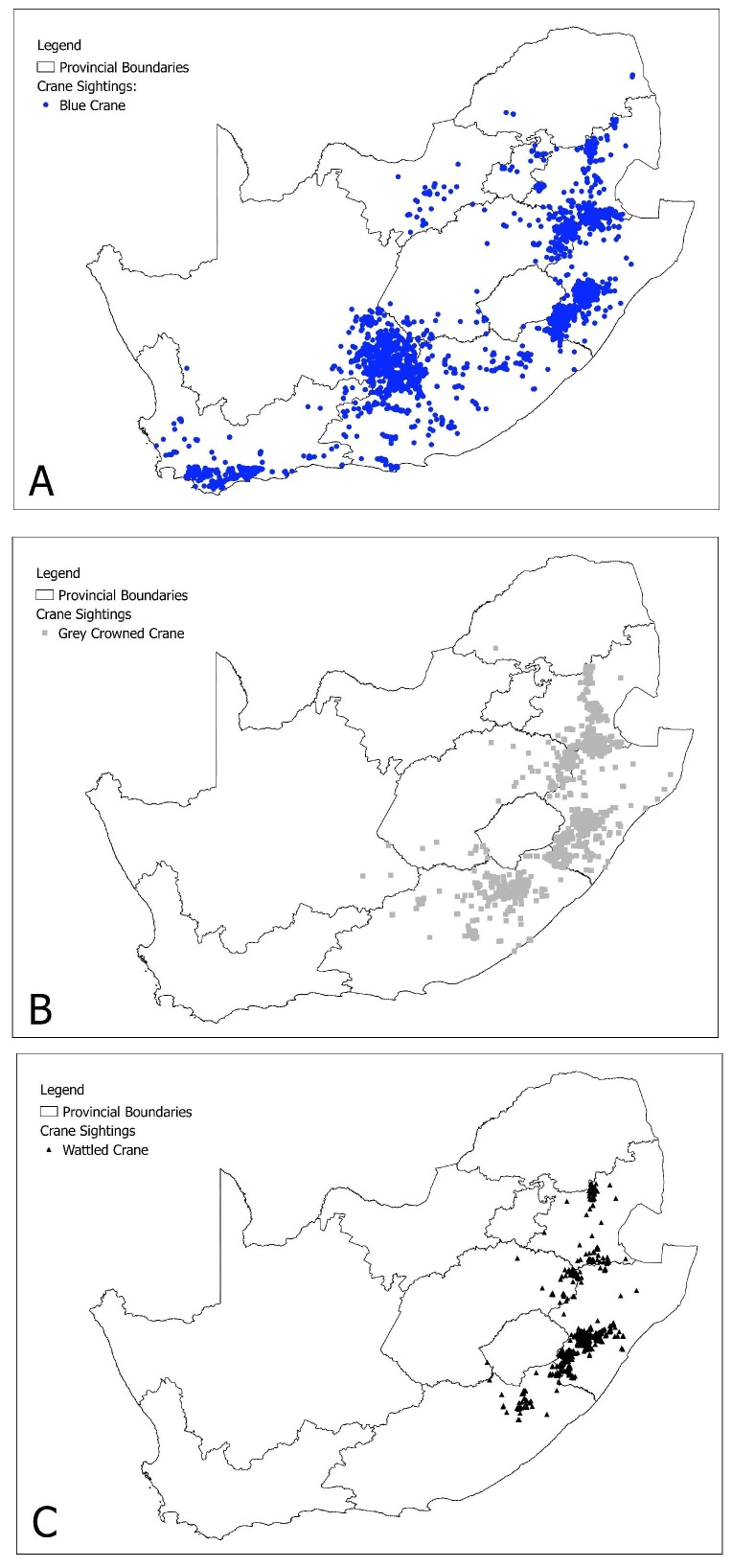
Sightings of three crane species in nine provinces in South Africa (1978-2014). A. Blue Crane. B. Grey Crowned Crane C. Wattled Crane.

**General spatial coverage**: The geographic range of the dataset covers five African countries: Kenya, Rwanda, South Africa, Uganda and Zambia (Table 1, Fig. 1). South Africa had the most recorded crane sightings (97.9%; Fig. 2), spread across all 9 provinces. Zambia had the second highest number of recorded crane sightings (0.8%) followed by Rwanda (0.6%), Uganda (0.4%) and Kenya (0.3%).

### Coordinates

-34.85 and 0.00 Latitude; 18.30 and 35.50 Longitude.

## Taxonomic coverage

### Description

**Figure 3.**
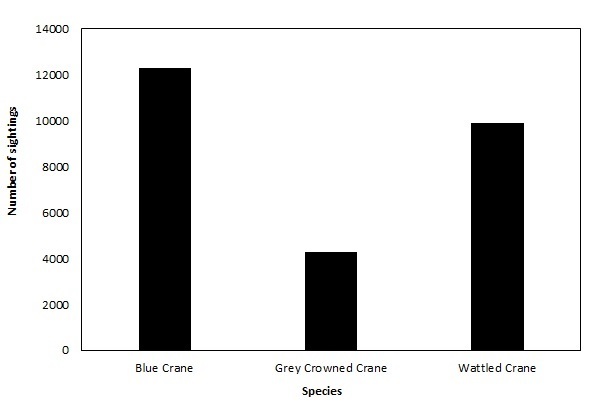
Taxonomic coverage of the dataset includes three crane species n = 26 403 (Family: Gruidae).

**General taxonomic coverage description**: The dataset contains three taxa in the family Gruidae belonging to the class Aves. Species that are recorded in the dataset are: the Blue Crane *Anthropoides paradiseus* (46.5% of the sightings), the Grey Crowned Crane *Balearica regulorum* (37.5% of the sightings) and the Wattled Crane *Bugeranus carunculatus* (16.1% of the sightings) (Fig. 3).

### Taxa included

**Table:** 

Rank	Scientific Name	
kingdom	Animalia	
phylum	Chordata	
class	Aves	
order	Gruiformes	
family	Gruidae	Cranes
species	Anthropoides paradiseus	Blue Crane
species	Balearica regulorum	Grey Crowned Crane
kingdom	Bugeranus carunculatus	Wattled Crane

## Temporal coverage

**Data range:** 1978 4 24 – 2014 12 12.

### Notes

**Figure 4.**
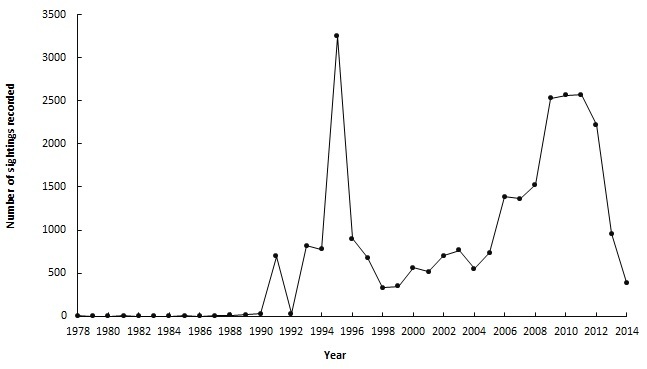
Temporal profile of the data collected between 1978 and 2014.

**General temporal coverage**: The temporal range of the records is between 1978 and 2014 (Fig. 4). No data were recorded for the years 1980, 1982 and 1984. During these periods there were no specific projects targeted at collecting sightings and as a result data were recorded on an ad hoc basis, often resulting in little or no data. Generally, the number of sightings per year fluctuates but there have been two peak data periods: 1994 – 1996 and 2008 – 2012 (Fig. 4). The most records were collected in 1995 with 3 249 recorded sightings, followed by 2 568 records in 2010. These peaks can be explained by the survey effort during this time. Between 1994 and 1996, the EWT started one project in KwaZulu-Natal and one in Mpumalanga, thus increasing the survey effort and therefore more sightings were recorded. In 2008, the ACCP received a Darwin Initiative project which focused intensely on monitoring, resulting in the second peak in data collection.

## Usage rights

### Use license

Open Data Commons Attribution License

## Data resources

### Data package title

﻿a﻿fri﻿cancranesightings

### Number of data sets

1

### Data set 1.

#### Data set name

EW﻿﻿T:﻿ African Crane Conservation Programme Sightings

#### Data format

Darwin Core Archive

#### Number of columns

26

#### Download URL

http://www.gbif.org/dataset/b9f3beb1-13c0-484e-886b-83e9062be37a

#### Data format version

Version 1

#### Description

The dataset is formatted according to Darwin Core standards (http://rs.tdwg.org/dwc/terms/) and the column labels and column descriptions are based on this standard.

**Data set 1.** 

Column label	Column description
occurrenceID	An identifier for the Occurrence, which is constructed from a combination of identifiers in the record that will most closely make the occurrenceID globally unique.
collectionCode	The name, acronym, coden, or initialism identifying the collection or data set from which the record was derived. In this ACCP is used, which is the acronym for the African Crane Conservation Programme of the International Crane Foundation / Endangered Wildlife Trust Partnership.
catalogNumber	A unique identifier for the record within the data set or collection.
Day	The integer day of the month on which the Event occurred.
Month	The ordinal month in which the Event occurred.
Year	The four-digit year in which the Event occurred, according to the Common Era Calendar.
Country	The name of the country in which the Location occurs.
stateProvince	The name of the next smaller administrative region than country (state, province, canton, department, region, etc.) in which the Location occurs.
Locality	The specific description of the place, frequently the name of a farm or protected area.
decimalLatitude	The geographic latitude (in decimal degrees, using the spatial reference system given in geodeticDatum) of the geographic center of a Location.
decimalLongitude	The geographic longitude (in decimal degrees, using the spatial reference system given in geodeticDatum) of the geographic center of a Location.
geodeticDatum	The ellipsoid, geodetic datum, or spatial reference system (SRS) upon which the geographic coordinates given in decimalLatitude and decimalLongitude as based. In this case, it is WGS84
locationRemarks	Comments or notes about the Location. In this case, information is given about the distance and direction of the birds from the observer, where recorded. Or alternatively, in some cases the records are at a quarter degree (or QDS) scale and Latitude and Longitude are the QDS centre.
georeferenceVerificationStatus	A categorical description of the extent to which the georeference has been verified to represent the best possible spatial description. In this case, georeferencing was verified by the data custodians, namely the Endangered Wildlife Trust
vernacularName	A common or vernacular name.
Genus	The full scientific name of the genus in which the taxon is classified.
scientificName	The full scientific name.
individualCount	The number of individuals represented present at the time of the Occurrence.
Lifestage	The age class or life stage of the biological individual(s) at the time the Occurrence was recorded.
behaviour	A description of the behavior shown by the subject at the time the Occurrence was recorded. These are in 5 categories: Feeding, Breeding, Roosting, Flying and Unknown
eventRemarks	Comments or notes about the Event. In this case, this column records whether birds were seen singly, in pairs or family groups or in flocks.
RecordedBy	A list of names of people, groups, or organizations responsible for recording the original Occurrence.
basisOfRecord	The specific nature of the data record, in this case all are HumanObservation
institutionCode	The name (or acronym) in use by the institution having custody of the object(s) or information referred to in the record.
ownerInstitutionCode	The name (or acronym) in use by the institution having ownership of the object(s) or information referred to in the record.
fieldNotes	The text of notes taken in the field about the Event.

## Figures and Tables

**Table 1.** Table 1: The spatial coverage of the dataset - Countries, Provinces and Districts

**Country**	**Region**	**Province**	**District**	**Total**
Kenya		Rift Valley		86
Rwanda		Northern	Burera	128
		Northern	Gicumbi	17
		Northern	Unknown	3
South Africa		Eastern Cape		6215
		Free State		1362
		Gauteng		55
		KwaZulu-Natal		10525
		Limpopo		11
		Mpumalanga		3950
		Northern Cape		2826
		North-West		43
		Western Cape		868
Uganda	Central Region		Lwengo	32
	Western Region		Bushenyi	10
	Western Region		Kabale	48
	Western Region		Mitooma	9
	Western Region		Ntungamo	8
	Western Region		Sheema	3
Zambia		Central	Chibombo	5
		Central	Mumbwa	61
		Southern	Monze	137
